# X-ray dynamical diffraction by quasi-monolayer graphene

**DOI:** 10.1038/s41598-023-43269-6

**Published:** 2023-09-24

**Authors:** Olena S. Skakunova, Stepan I. Olikhovskii, Taras M. Radchenko, Svitlana V. Lizunova, Tetyana P. Vladimirova, Vyacheslav V. Lizunov

**Affiliations:** grid.435300.1 0000 0004 0482 7152 G. V. Kurdyumov Institute for Metal Physics of the N.A.S. of Ukraine, Kyiv, Ukraine

**Keywords:** X-rays, Surfaces, interfaces and thin films, Mechanical and structural properties and devices

## Abstract

We study the processes of dynamical diffraction of the plane X-ray waves on the graphene film/SiC substrate system in the case of the Bragg diffraction geometry. The statistical dynamical theory of X-ray diffraction in imperfect crystals is applied to the case of real quasi-two-dimensional systems. The necessity of the taking into account of the variability of the lattice parameter of multilayer graphene, as well as the influence of thickness on the thermal Debye–Waller factor at the calculation of the complex structural factors and Fourier components of polarizability, is demonstrated. It is shown that the change of the structural characteristics of the 3-layer graphene/substrate system, as well as its strained state, leads to a significant change in the diffraction profiles, which makes it possible to determine the characteristics by the X-ray diffraction method.

## Introduction

The development of modern physical materials science is inseparably linked with the determination of structural characteristics of materials. This is due to the material structure mainly determines their most important physical and exploitation properties. At the same time, the main characteristics of materials (mechanical, electrical, magnetic, optical, etc.) as a rule strongly depend on their defect structure. Therefore, the problem of creation of the materials with predetermined properties is closely related to the improvement of already known and the development of new methods of characterization of the material structure.

Among other perspective materials, graphene has unique mechanical, electrical properties and can be used in many areas. At the same time, the properties of the graphene film depend significantly on the method of its preparation. In particular, chemical vapor deposition (CVD)^1–3^ and SiC substrate annealing^4–6^ are effective methods for large-scale graphene production. Since the properties of graphene depend, in particular, on its structural parameters, it is important to carry out an accurate quantitative characterization of the structure of the graphene film on the substrate.

For structural characterization of thin films, multilayer systems, superlattices, etc. nondestructive diffraction methods are widely used^7–11^. The effectiveness of any diffraction method is largely determined by the analytical expressions that provide an adequate description of the scattering pattern. Both the kinematical approximation and the dynamical theory are used in the X-ray characterization of crystalline materials.

The kinematical description gives a simple expression for the scattering amplitude, which, in fact, is the Fourier transform of the electron density of the crystal. However, the kinematical approximation is violated for crystals whose dimensions are comparable with the extinction length. In this case, dynamical effects (multiple scattering effects), namely the interaction between diffracted and transmitted waves, together with refraction and absorption, can become significant. At the same time, partially dynamical effects can be manifested even for ‘thin’ crystals, so their rigorous description requires the use of dynamical scattering theory.

The dynamical scattering theory attracted an attention of researchers and got a development almost immediately and intensively after the discovery of the radiation diffraction on crystal structures. However, only since 1960s, when methods for growing almost perfect single crystals had developed, a rapid growth of a series of studies related to the dynamical diffraction was additionally motivated and stimulated. Particularly, the dynamical theories based on the Ewald–Bethe–Laue approach^12^ and optical potential method^13–15^ to describe the Borrmann effect were developed.

Then, the Takagi–Taupin equations became the fundamental base of a statistical dynamical diffraction theory proposed in Refs.^16,17^ for the case of incident spherical wave diffracted on the mosaic crystal. This theory was repeatedly verified experimentally (see, e.g., Ref.^18^).

The case of incident plane waves was considered for describing the angular distributions of the diffracted X-ray (see, e.g.^19^). Just that approach was used to calculate the distributions of X-ray intensity scattered by imperfect crystals^20–24^.

At the same time, Holý proposed another variant of dynamical theory based on the optical coherence formalism^25–28^ and applied this approach for determining the parameters of the single crystals’ microdefects^29–31^. In addition, the distorted wave Born approximation was used for calculation of scattering patterns for the case when the microdefects are contained in the thin crystalline layers^32^.

In articles^33–38^, authors also pay attention to the dynamical effects in the diffuse scattering theory. For this purpose, the statistical dynamical theory of X-ray diffraction by imperfect single crystals with randomly distributed microdefects was used^37–40^. This theory is based on the Ewald–Bethe–Laue approach, where the scattering problem is considered in the 3*D* momentum space. It makes possible establishing direct analytical formulas between Fourier components of the fluctuating part of crystal polarizability and defect characteristics. It is precisely this theory that uses in presented work.

Thus, the most studies and therefore obtained results describe mainly the bulk crystals. There is still a lack in the studying the quasi-two-dimensional (2*D*) systems, including the firstly know 2*D* material—graphene, which contributes to the motivation of the study. The characterization of quasi-monolayer structures by the methods of dynamical diffractometry has the specific and certain features. The scope of this article deals with extension of the statistical dynamical theory of X-ray diffraction in imperfect crystals to the case of realistic quasi-monolayer systems with inhomogeneous strain fields and microdefects.

## Diffraction parameters

We consider the diffraction of the plane X-ray waves in the case of the Bragg diffraction geometry (the case of reflected radiation). According to the optical potential method, the polarizability of the crystal is represented as a complex quantity^41,42^:

1 $$ \upchi \left( {\mathbf{r}} \right) = \upchi_{{\text{r}}} \left( {\mathbf{r}} \right) + i\upchi_{{\text{i}}} \left( {\mathbf{r}} \right). $$

Accordingly, the Fourier component of polarizability can be represented as follows:

2 $$ \upchi_{{\mathbf{H}}} = \upchi_{{{\text{r}}{\mathbf{H}}}} + i\upchi_{{{\text{i}}{\mathbf{H}}}} . $$

In the case of a non-centrosymmetrical crystal, the Fourier components χ_r**H**_ and χ_i**H**_ are also complex quantities proportional to the corresponding structural factors^43^:

3 $$ \upchi_{{{\text{r}}{\mathbf{H}}}} = - \Gamma F_{{{\text{r}}{\mathbf{H}}}} ,\upchi_{{{\text{i}}{\mathbf{H}}}} = - \Gamma F_{{{\text{i}}{\mathbf{H}}}} , $$

4 $$ F_{{{\text{r}}{\mathbf{H}}}} = \sum\limits_{j} {f_{{\text{r}}} \exp (i\varphi_{j} )} ,\;F_{{{\text{i}}{\mathbf{H}}}} = \sum\limits_{j} {f_{{\text{i}}} \exp (i\varphi_{j} )} , $$

where Γ = *r*_*e*_ λ^2^/π*v*_c_, *r*_*e*_ is classical electron radius, λ is wavelength of X-ray radiation, *v*_c_ = (3)^1/2^*a*^2^*c*/2 is unit cell volume, *a* i *c*—hexagonal lattice parameters, φ_*j*_ = 2π (*hx*_*j*_ + *ky*_*j*_ + *lz*_*j*_), *h*, *k*, *l* are Miller indices. The summation in (4) is carried out on the coordinates (*x*_*j*_, *y*_*j*_, *z*_*j*_) of the atoms in the unit cell, *f*_r_ and *f*_i_ are the real and imaginary parts of the atomic form factor:

5 $$ f_{{\text{r}}} = (f_{0} + \Delta f)\exp ( - M),\;f = \frac{1}{{2{\uplambda }r_{e} }}{\upsigma }_{{\text{a}}}^{{\mathbf{H}}} \exp ( - M), $$

where *f*_0_ and ∆*f* are the atomic form factors at absolute zero temperature and the dispersion correction respectively, and exp(−*M*) is the thermal Debye–Waller factor. The atomic photoelectric absorption cross section has the form^44,45^:

6 $$ {\upsigma }_{{\text{a}}}^{{\mathbf{H}}} = C\frac{{\upmu }}{{\uprho }}\frac{{M_{{\text{a}}} }}{{N_{{\text{A}}} }}, $$

where (μ/ρ) is mass absorption coefficient, which was taken from Ref.^46^, *M*_a_ is atomic mass, *N*_A_ is Avogadro constant, *C* = 1 or cos(2θ_B_) is polarization factor, respectively, for σ- and π-polarization,

$$ {\uptheta }_{{\text{B}}} = \arcsin \left( {\frac{{\uplambda }}{2a}\sqrt {\frac{{4(h^{2} + hk + k^{2} )}}{3} + \frac{{l^{2} a^{2} }}{{c^{2} }}} } \right), $$

is Bragg angle.

An interpolation formula was used to calculate the atomic form factors^47^:

7 $$ f_{0} (S) = \sum\limits_{i = 1}^{5} {a_{i} e^{{ - b_{i} S^{2} }} } + c, $$

where *a*_*i*_, *b*_*i*_, *c*_*i*_ are tabulated parameters, *S* = sinθ_B_/λ. Dispersion corrections ∆*f* were taken from work^46^.

The exponent of thermal Debye–Waller factor in expression (5) was calculated using the formula that takes into account its anisotropy^48^:

8 $$ M = H_{{\text{p}}}^{{2}} U_{{\text{p}}}^{2} + H_{z}^{2} U_{z}^{2} , $$

where $H_{{\text{p}}}^{{2}} = H_{x}^{2} + H_{y}^{2} ,$ *H*_p_ is the planar component of the scattering vector and *H*_*z*_ is its *z*-component. The root-mean-square displacements of lattice atoms $U_{{\text{p}}}^{2}$ and $U_{z}^{2}$ are taken from work^44^.

## X-ray diffraction by quasi-monolayer systems

As mentioned, one of the effective methods of preparation graphene is its growth by annealing the SiC substrate. The analytical expressions for the intensity of diffracted X-ray radiation on the graphene film/SiC substrate system were used the recurrence relations between the coherent components of the amplitude reflection coefficients of adjacent layers^49^, which were obtained within the framework of the generalized dynamical theory of scattering in imperfect single crystals with randomly distributed defects^39^.

The coherent component of the reflectivity of a multilayer crystal system, which consists of *M* layers and a substrate, in the case of the Bragg diffraction geometry is described as follows:

9 $$ R_{{{\text{coh}}}} {(}\Delta {\uptheta )} = \left| {R_{{\text{M}}} {(}\Delta {\uptheta )}} \right|^{2} . $$

*R*_M_(∆θ) is calculated using the recurrence relation between the amplitude reflection coefficients of two systems consisting of *M* and (*M* − 1) layers^49^:

10 $$ R_{j} = \frac{{r_{j} + R_{j - 1} (e_{j}^{ - 1} t_{j}^{2} - \varsigma_{j}^{ - 1} r_{j}^{2} )}}{{1 - \varsigma_{j}^{ - 1} r_{j} R_{j - 1} }}, $$

where *j* = 1, …, *M*, *R*_0_ ≡ *r*_0_, ∆θ is angular deviation of the crystal from the Bragg angle. *r*_*j*_ and *t*_*j*_ are the amplitude coefficients of reflection and transmission of the *j*th layer, respectively:

11 $$ r_{j} = \varsigma_{j} b^{1/2} \frac{{e^{{ - iK\Delta_{1}^{j} d_{j} }} - e^{{ - iK\Delta_{2}^{j} d_{j} }} }}{{B_{1}^{j} - B_{2}^{j} }}, $$

12 $$ t_{j} = e_{j} \frac{{c_{1}^{j} - c_{2}^{j} }}{{B_{1}^{j} - B_{2}^{j} }}, $$

13 $$ e_{j} = \exp [ - iK(\Delta_{1}^{j} + \Delta_{2}^{j} )d_{j} ], $$

14 $$ B_{{\updelta }}^{j} = c_{j}^{{\updelta }} e^{{ - iK\Delta_{{\updelta }}^{j} d_{j} }} ,\;c_{j}^{\delta } = (\varsigma_{j} b)^{1/2} (y_{i} + ( - 1)^{\delta } \sqrt {y_{i}^{2} - 1} ),\;\varsigma_{j} = \sqrt {\frac{{\upchi_{{{\mathbf{H}}j}} }}{{\upchi_{{ - {\mathbf{H}}j}} }}} , $$

where the index *j* indicates the connection of the corresponding value with the *j*th layer, *b* = γ_0_/|γ_**H**_**|** is the parameter of the diffraction asymmetry, γ_0_ and γ_**H**_ are the direction cosines of the wavevectors of the incident and diffracted plane waves, respectively, **H** is the reciprocal-lattice vector, *K* = 2π/λ, *d*_*j*_ is the thickness of *j*th layer, δ = 1, 2. The accommodations of the strong Bragg wavevectors in the *j*th layer, ${\mathbf{K}}_{0j}^{\delta }$ and ${\mathbf{K}}_{{{\mathbf{H}}j}}^{\delta } ,$, are described as follows:

$$ \Delta_{{\updelta }}^{j} = \frac{1}{{2{\upgamma }_{{0}} }}{\upchi }_{0j} + \frac{{\uplambda }}{{2\Lambda_{j} }}(y_{i} + ( - 1)^{{\updelta }} \sqrt {y_{i}^{2} - 1} ),\; $$

15 $$ \Lambda_{j} = {\uplambda }({\upgamma }_{{0}} \left| {{\upgamma }_{{\mathbf{H}}} } \right|)^{1/2} /{\upsigma }_{j} ,\;{\upsigma }_{j}^{2} = C^{2} {\upchi }_{{{\mathbf{H}}j}} {\upchi }_{{ - {\mathbf{H}}j}} . $$

The normalized angular deviation *y*_*j*_ in formulas (14) and (15) is determined by the deviation ∆**H** of the reciprocal-lattice vector of the substrate **H** ≡ **H**_0_ (*j* = 0) due to the sample rotation and by the deviations ∆**H**_*j*_ of the reciprocal-lattice vector **H**_*j*_ due to the average strain caused by defects or chemical composition of *j*th layer:

16 $$ y_{j} = ({\upalpha }_{j} - {\upalpha }_{0j} )\sqrt b /{\upsigma }_{j} , $$

17 $$ \upalpha_{j} = - (\Delta \uptheta { + }\Delta \uptheta_{j} )\sin (2\uptheta_{{\text{B}}} ),\;\upalpha_{0j} = \left( {1 + \frac{1}{b}} \right)\frac{{\upchi_{0j} }}{2}, $$

18 $$ \Delta {\uptheta }_{j} = (\varepsilon_{ \bot }^{j} \cos^{2} \psi + \varepsilon_{\parallel }^{j} \sin^{2} \psi )\tan \uptheta_{{\text{B}}} + {\text{sgn}} (1 - b)((\varepsilon_{ \bot }^{j} - \varepsilon_{\parallel }^{j} )\sin \psi \cos \psi , $$

where ∆θ_*j*_ is the angular deviation of the *j*th layer from the substrate orientation due to strain, ψ is the angle between the crystal surface and the reflective planes. The parallel and normal strain components in the *j*th layer are described as follows:

19 $$ \varepsilon_{\parallel }^{j} = \frac{{a_{j} - a_{0} }}{{a_{0} }},\;\varepsilon_{ \bot }^{j} = \frac{{c_{j} l_{0} }}{{c_{0} l_{j} }} - 1, $$

20 $$ a_{j} = a_{j}^{0} - R(a_{j}^{0} - a_{0} ),\;c_{j} = c_{j}^{0} \left( {DR\frac{{a_{j}^{0} - a_{0} }}{{a_{j}^{0} }}} \right) + 1, $$

where *R* is a tension parameter (0 ≤ *R* ≤ 1) that represents or can be associated with a strain strength or percentage, *D* = 2*c*_13_/*c*_33_ is distortion factor, *a*_0_, *c*_0_ and *a*_*j*_, *c*_*j*_ are lattice parameters for the substrate and layer, respectively, ${a}_{j}^{0}$ and ${c}_{j}^{0}$ are lattice parameters of the layer in the free state, *l*_0_ and *l*_*j*_ are the components of Miller indices for substrate and layer, respectively.

## Results of numerical calculations

**Figure 1:**
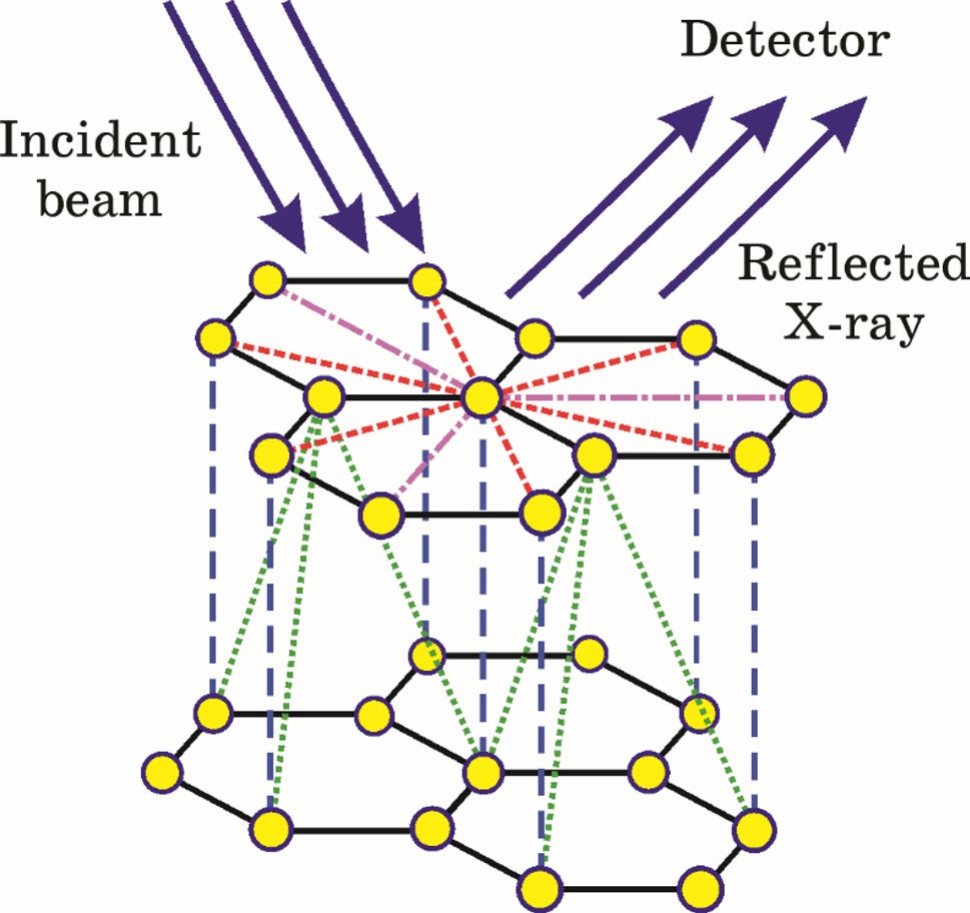
The geometry of X-ray diffraction on multilayer graphene. Two layers (AB) of Bernal-stacked multilayer graphene and incident and reflected beams are presented.

At first, using formulas (3)–(8), the X-ray diffraction parameters of multilayer graphene with the packing of atoms of the A–B–A type (so-called Bernal-stacked multilayer graphene) were obtained. The geometry of diffraction experiment is presented on Fig. 1.

In the article^4^, the parameter β was used for to describe of the structure of graphene layers. This parameter characterizes an occupancy of graphene layers (its value belongs the range [0, 1]). Thereby the used model has two fitting parameters for each layer: interlayer spacing and occupancy of graphene layer. In our calculation, we consider graphene layers as the completely occupied (β = 1).

It should be noted, that we characterize a quasi-two-dimensional object, which is such according to its physical properties. However, here it is assumed that in the plane, parallel to the surface of the system, all layers (graphene, buffer and substrate) are homogeneous. It is due to this that the diffraction problem can be considered as one-dimensional along the axis, which is perpendicular to the surface of the system, which is inhomogeneous in this direction.

**Figure 2:**
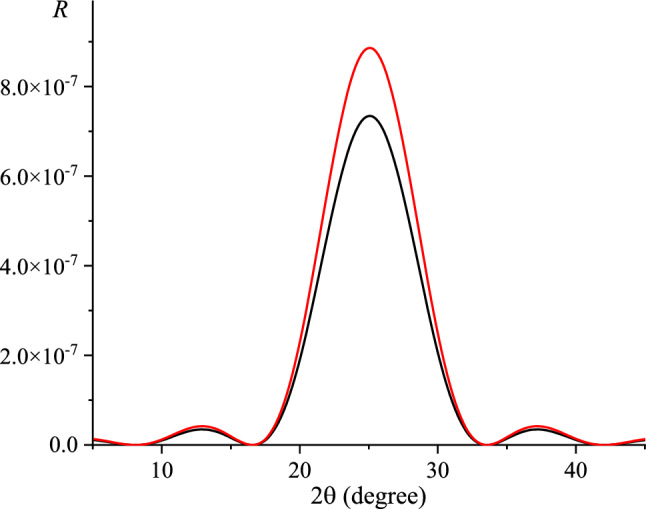
Calculated RCs of 3-layer graphene (reflex (002), Cu*K*_α1_) with (black line) and without (red line) taking into account the thickness dependence of the root-mean-square atomic displacement at the calculation of diffraction parameters.

Formula (8) takes into account the dependence of the root-mean-square displacement of lattice atoms $U_{{\text{Z}}}^{2}$ on the film thickness^44^. It should be noted that the use of root-mean-square displacements for films of small thicknesses (about several atomic layers) corresponding to a bulk sample would lead to significant errors in the calculation of the thermal Debye–Waller factor and the Fourier components of the crystal polarizability and, as a result, to errors at the calculation of the reflection curves (RCs). This fact is illustrated Fig. 2, which shows RCs with and without taking into account the thickness dependence of the root-mean-square displacement of atoms at the calculation of diffraction parameters. The maximum difference between the curves is 20%.

**Figure 3:**
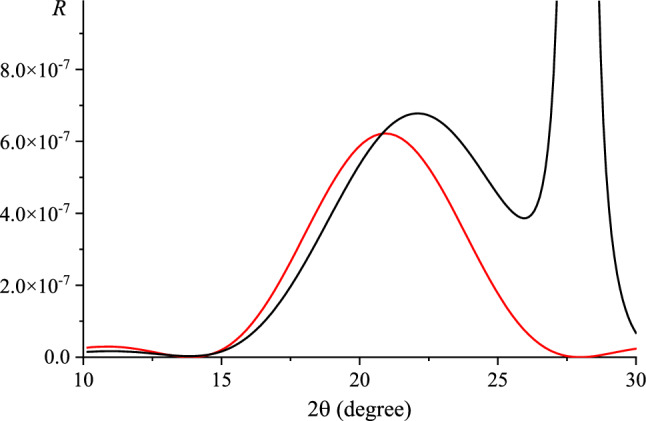
The calculated RCs of 3-layer graphene (reflex (002), radiation energy is 10.2 keV) with (black line) and without (red line) taking into account the presence of 6H-SiC substrate.

Figure 3 shows the calculated RCs also taking into account the presence of a substrate for the (002) reflex of 3-layer graphene for radiation with an energy of 10.2 keV. The interlayer distance in a graphene film depends on the conditions of its preparation, the number of layers, and the number of the layer from the substrate^4,50,51^. So, as initial values of lattice parameters were used lattice parameters of graphite, namely *a* = 0.246 nm and *c* = 0.6708 nm. The Fourier components of the polarizability were calculated for these lattice parameters.

Figure 3 shows, that the taking into account a substrate at the calculation of RCs leads the significantly shift of the diffraction peak from the graphene layers (Fig. 3, solid line).

**Figure 4:**
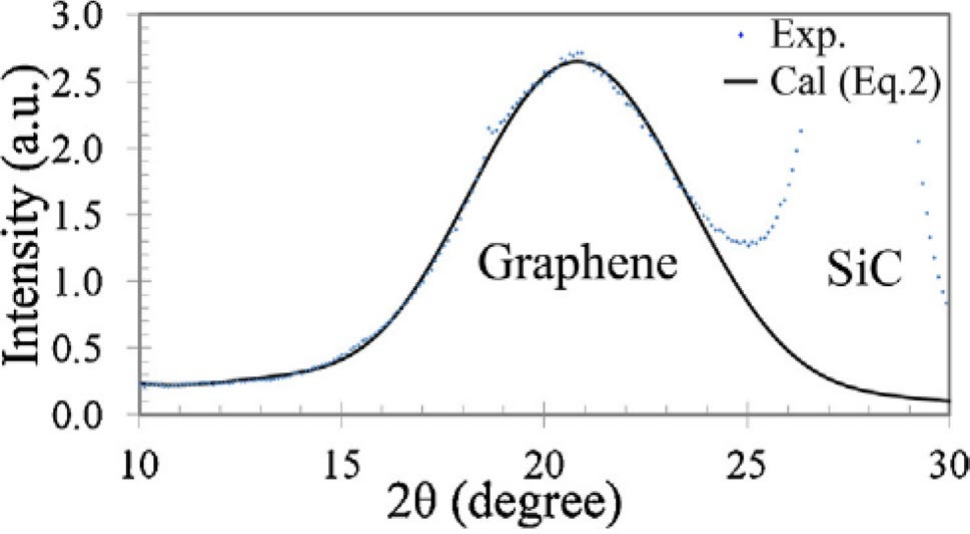
Experimental X-ray diffraction RC (markers) of a graphene film grown on a SiC (0001) surface^4^. The solid line is the result of the calculation by authors of the paper^4^. The interlayer distance *d* in the film was determined to be 3.30 ± 0.05 Å.

For comparison, Fig. 4 shows the experimentally obtained RC for the system multilayer graphene/substrate 6H-SiC (0001)^4^. It can be seen that the results of the calculations of this work and the experimental data are in good agreement to each other.

It should be noted, that one of the stages of creating multilayer graphene films on a SiC substrate is the formation of a buffer layer (a hexagonal carbon layer)^52^. This layer is not yet graphene, because it is partially connected to the substrate due to the presence of *sp*^3^-hybridized regions. According to Ref.^53^, the interplanar distance between the buffer layer and the SiC substrate is *d* = 0.216 nm.

**Figure 5:**
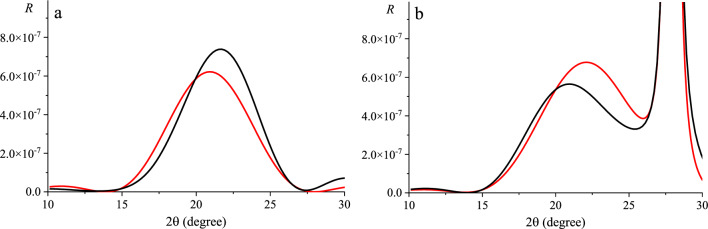
Calculated RCs for graphene (reflex (002), radiation energy is 10.2 keV) with the considering (black lines) and without the considering (red lines) the presence of the buffer layer: 3-layer graphene (**a**), system 3-layer graphene/6H-SiC substrate (**b**).

Figure 5 shows the change of RCs at the taking into account the presence of the buffer layer for both cases presented in Fig. 3. It should be noted that the presence of the buffer layer for a system consisting only of multilayer graphene (without a substrate) (Fig. 5a) leads to an increase in the height of the peak and a shift of its maximum to the right. At the same time, the intensity of the peak for multilayer graphene on the substrate (Fig. 5b) with taking into account the presence of the buffer layer decreases and shifts to the left.

**Figure 6:**
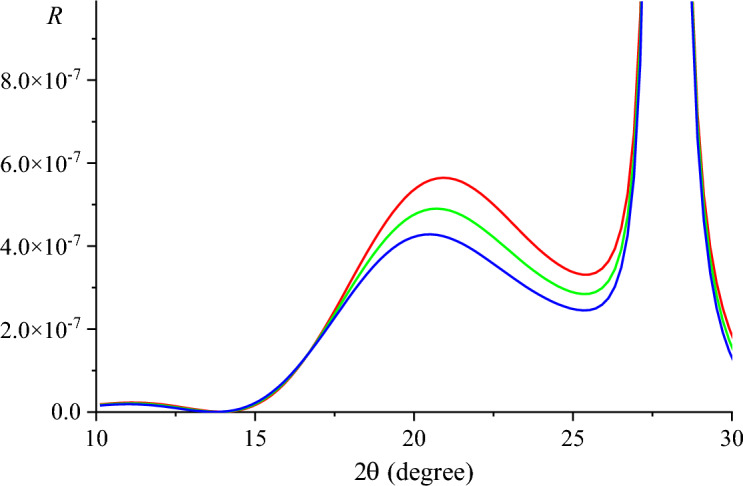
Calculated RCs of the 3-layer graphene/6H-SiC substrate (reflex (002), radiation energy is 10.2 keV) with different stress parameters of the graphene layers: *R* = 0 (red line), 0.5 (green line), 1 (blue line).

One of the remarkable properties of graphene—known as a strongest material ever tested—is its high flexibility along with strength. Such unique mechanical properties caused the development of the relatively new research direction in the physics of graphene and related 2*D* materials known as ‘straintronics’^54–58^ and ‘twistronics’^59–61^. Figure 6 shows RCs with taking into account the strain of the graphene layers. We considered the case when the corresponding translation vectors of the layer are rotated by 30° comparative to the substrate. Accordingly, *a*_0_ in formulas (19) and (20) was replaced by *a*_0_cos30°. Poisson’s ratios for multilayer graphene ν_i_ = 0.15 (in-plane) and ν_o_ = − 0.09 (out-of-plane) were taken from the work^62^.

It can be seen that the change in the strain state of the 3-layer graphene/substrate system leads to a change in the shape of RCs.

## Conclusions

In this article, the statistical dynamical theory of X-ray diffraction of the plane X-ray waves in imperfect crystals is applied to the case of real quasi-two-dimensional systems. The case of the Bragg diffraction geometry is considered. The necessity of the taking into account of the variability of the lattice parameter of multilayer graphene, as well as the influence of thickness on the thermal Debye–Waller factor at the calculation of the complex structural factors and Fourier components of polarizability, is demonstrated. It is shown that the change of the structural characteristics of the 3-layer graphene/substrate system, as well as its strained state, leads to a significant change in the diffraction profiles, which makes it possible to determine the characteristics by the X-ray diffraction method.

The X-ray diffraction characteristics are sensitive to the structural strains independently on their types (stretching, shearing, twisting, etc.). Therefore, the XRD method can act as a powerful or at least additional tool for detecting of any responses in the structural changes occurring in graphene and other currently discovered 2*D* materials. Particularly, such the sensitivity can contribute to the overcoming the challenges dealing with detecting so-called magic angles in the twisted 2*D* material offering a method to modify its electronic properties.

The proposed dynamical approach, as an additional tool, allows describing correctly the diffraction profiles from such structures in the region of the Bragg peak from the substrate and its immediate vicinity. Due to this, we can expect to improve the reliability of the diffraction characterization and possibly obtain additional accurate information about the structure of the system, because the proposed method allows us to take into account the influence of the substrate and its defects on the formation of the total diffraction pattern.

## Data Availability

The data that support the findings of this study available from the corresponding author on reasonable request.
